# Cocaine-Like Discriminative Stimulus Effects of Mephedrone and Naphyrone in Mice

**DOI:** 10.4303/jdar/236009

**Published:** 2016-12-31

**Authors:** Brenda M. Gannon, William E. Fantegrossi

**Affiliations:** Department of Pharmacology and Toxicology, College of Medicine, University of Arkansas for Medical Sciences, Little Rock, AR 72205, USA

**Keywords:** bath salts, cathinones, drug discrimination

## Abstract

**Background:**

In recent years, commercial *bath salts* products containing synthetic cathinone analogues have emerged as illicit drugs of abuse. These cathinones are structurally similar to the psychostimulants 3,4-methylenedioxymethamphetamine (MDMA) and methamphetamine (METH), and produce their effects via interactions with monoamine transporters, where smaller compounds (e.g., mephedrone) are amphetamine-like monoamine releasers, while the structurally larger compounds (e.g., naphyrone) are cocaine-like monoamine reuptake inhibitors. Individual cathinones also differ from one another with respect to selectivity among the three monoamine transporters.

**Statement of purpose of study:**

This study was designed to assess the cocaine-like interoceptive effects of synthetic cathinone analogues functioning as passive monoamine reuptake inhibitors (naphyrone) or as releasers (mephedrone) in mice in order to compare effectiveness (degree of substitution) and potency with positive control psychostimulants cocaine, METH, and MDMA.

**Procedures:**

In the present study, mice were trained to discriminate 10 mg/kg cocaine from saline, and substitutions with METH, MDMA, mephedrone, naphyrone, and morphine were performed.

**Main findings:**

Mice reliably discriminated the cocaine training dose from saline, and METH, MDMA, mephedrone, and naphyrone all elicited full cocaine-like responding, while morphine did not. Potency differences were observed such that METH was most potent, while mephedrone, cocaine, MDMA, and naphyrone exhibited roughly equivalent potency.

**Principal conclusions:**

These data confirm that interaction with DAT is an important component of cocaine-like discriminative stimulus effects, and suggest that synthetic cathinones likely elicit psychostimulant-like abuse-related effects.

## 1. Introduction

Synthetic analogues of cathinone—a naturally-occurring psychostimulant derived from the *khat* plant—have emerged as psychostimulant drugs of abuse in *bath salt* preparations. Individual constituents in these preparations are structurally similar to psychostimulants methamphetamine (METH) and 3,4-methylenedioxymethamphetamine (MDMA). Recently, the United Nations reported roughly 25% of all new psychoactive substances are synthetic cathinones [1], and 13 of these cathinone analogues have been classified by the US Drug Enforcement Administration as Schedule I. 4-Methylmethcathinone (mephedrone) and naphthylpyrovalerone (naphyrone) are two common *bath salts* constituents which have been reclassified as Schedule I compounds [1,2,3]. The mechanisms by which these two compounds produce their effects differ from one another, such that mephedrone not only inhibits monoamine uptake through the monoamine transporters but also stimulates the release of monoamines at the transporters, while naphyrone inhibits monoaminergic reuptake at the transporters without stimulating monoamine release [4].

Drug discrimination is an in vivo assay utilized to characterize and screen centrally active compounds [5,6]. Previously, our lab trained mice to discriminate another common *bath salt* constituent, 3,4-methylenedioxypyrovalerone (MDPV), from saline and reported that METH and MDMA both produced MDPV-like responding; however neither the synthetic cannabinoid JWH-018 nor the *μ* opioid receptor agonist morphine elicited MDPV-appropriate responding [7]. In a second group of mice trained to discriminate 10 mg/kg cocaine from saline, we demonstrated that MDPV dose-dependently produced cocaine-appropriate responding [8], a finding consistent with studies conducted in rats [9] and nonhuman primates [10].

Cocaine is the prototypical abused psychostimulant that inhibits monoamine uptake without stimulating the release of monoamines. Since we have previously shown that METH and MDMA produced MDPV-like responding (but morphine did not), and that MDPV produced cocaine-like responding, we hypothesized that METH and MDMA (but not morphine) would produce dose-dependent increases in cocaine-appropriate responding in mice trained to discriminate cocaine from saline. More importantly, because compounds within the same drug class as the training drug tend to produce similar levels of drug-appropriate responding in drug discrimination, we also hypothesized the synthetic cathinones mephedrone and naphyrone would produce dose-dependent increases in cocaine-appropriate responding, and aimed to compare the relative potencies of each drug to produce cocaine-appropriate responding.

## 2. Materials and methods

### 2.1. Animals

All studies were carried out in accordance with the Guide for Care and Use of Laboratory Animals as adopted and promulgated by the National Institutes of Health. The Institutional Animal Care and Use Committee at the University of Arkansas for Medical Sciences approved all of the experimental protocols. Adult male NIH Swiss mice (Harlan Laboratories, Indianapolis, IN, USA) weighing 20–25 g on delivery were housed (15.24 × 25.40 × 12.70 cm^3^) in a temperature-controlled room in an Association for Assessment and Accreditation of Laboratory Animal Care-accredited animal facility. Room conditions were maintained at 22 ± 2 °C and 45–50% humidity, with lights set to a 12-hour light/dark cycle. Mice were drug naïve prior to training and were fed Lab Diet rodent chow (Laboratory Rodent Diet no. 5001, PMI Feeds, St Louis, MO, USA). Mice were food restricted throughout all studies to maintain weights at approximately 30 g. Appropriate supplemental feedings occurred after the completion of daily behavioral sessions.

### 2.2. Procedures

#### 2.2.1. Drug discrimination

##### General methods

Mice (*n* = 5) were trained to discriminate 10 mg/kg cocaine from saline in standard operant chambers for mice that were individually enclosed in larger lightproof Malaguard sound-attenuating cubicles (MED Associates, St. Albans, VT, USA). All mice were previously used to assess the cocaine-like discriminative stimulus effects of the synthetic cathinone 3,4-methylenedioxypyrovalerone (MDPV) and its enantiomers in studies described in [8].

##### Substitution testing

All experimental procedures were previously described in [8]. Briefly, discriminative control was established with cocaine, and substitution tests were conducted twice per week in each animal so long as performance did not fall below the criterion level of 80% injection-appropriate responding in any one of the previous two training sessions. Test sessions were conducted under extinction conditions and terminated after completion of a fixed ratio (FR) 10 on either lever or after 5 min if the FR10 was not met. Saline substitution sessions were conducted to ensure discriminative performance was maintained and to obtain baseline response rates against which to compare the effects of all test compounds. Doses of each test compound were presented in random order, and after all doses of a compound were tested, another compound was tested. In this manner, cocaine, METH, MDMA, mephedrone, naphyrone, morphine, and saline were tested in all mice.

### 2.3. Data analysis

Graphical presentation of all data depict mean *±*SEM. Response distribution data are expressed as percent cocaine-appropriate responding, which is the number of responses emitted on the cocaine-trained lever as a percentage of the total number of responses emitted. Response rate is the total number of responses divided by the time elapsed (in seconds) during the test session. Full generalization was said to occur if the group mean was significantly different (via one way ANOVA, followed by pairwise comparisons using the Holm-Sidak method) from saline and *>* 80% of the total responses were made on the cocaine-appropriate lever. Response rates were considered significantly different from control if the group mean was significantly different (via one way ANOVA, followed by pairwise comparisons using the Holm-Sidak method) from rates during the saline test session.

Potency data represent the mean ED_50_ *±* 95% confidence intervals. ED_50_ values were considered statistically different in cases where the confidence intervals were nonoverlapping. SigmaPlot 11 software (Systat Software, San Jose, CA, USA) was used to generate figures, interpolate ED_50_ values, and conduct statistical analyses.

### 2.4. Drugs

Morphine, cocaine, MDMA, and METH were obtained from the NIDA Drug Supply Program. Mephedrone and naphyrone were purchased from Cayman Chemical in Ann Arbor, MI, USA. All compounds were weighed as salts and dissolved in 0.9% physiological saline. Injections were administered intraperitoneally (IP) at a volume of 0.1 cc/10 g. Saline and all other experimental supplies were obtained from standard commercial sources.

## 3. Results

**Figure 1:**
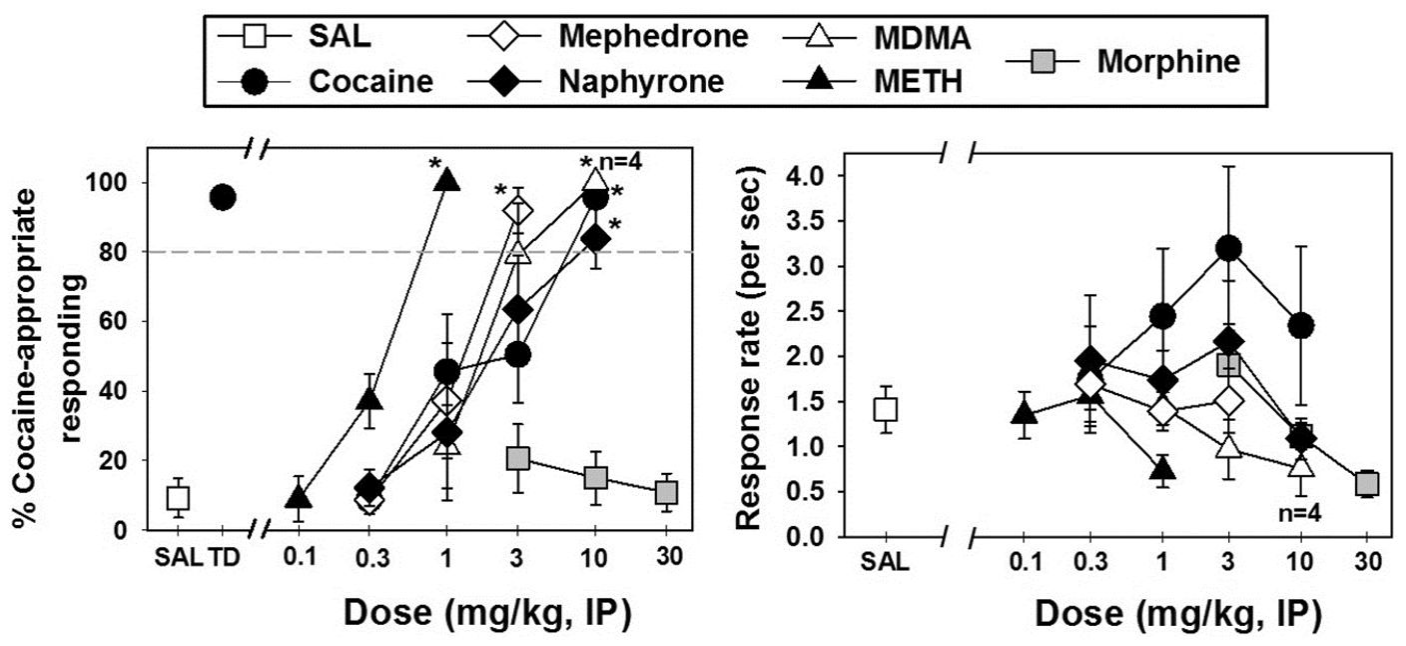
*(Left panel)* Discriminative stimulus effects of cocaine, mephedrone, naphyrone, *MDMA*, METH, and morphine in mice trained to discriminate 10 mg/kg cocaine from saline. *Abscissa*: SAL represents test injection of saline and TD represents administration of the cocaine training dose. Numbers refer to doses of drugs during substitution sessions, expressed as milligram per kilogram on a log scale. *Ordinate:* percent of total responses emitted on the cocaine-appropriate lever. *(Right panel)* Response rates following administration of saline, cocaine, mephedrone, naphyrone, *MDMA*, METH, or morphine during substitution sessions. The abscissa is as described above. *Ordinate*: response rates, expressed as lever presses per second. Asterisks adjacent to points indicate generalization at this dose. Errors bars depict means *±*SEM. The “*n* = 4” designation indicates only four mice were included in the data point (responding was suppressed in one mouse at 10 mg/kg MDMA).

Mice reliably learned to discriminate 10 mg/kg cocaine (black circle, “TD”) from saline (white square, “SAL”) (Figure 1, left panel). During training sessions, mice predominantly responded on the saline lever when administered saline and responded almost exclusively on the cocaine lever when 10 mg/kg cocaine was administered. Substitution test sessions with cocaine (black circles, Figure 1, left panel) resulted in dose-dependent increases in cocaine-lever responses, and the training dose (10 mg/kg) produced *>* 80% cocaine-appropriate responding and was significantly different from the discriminative responding elicited by saline (*t* = 12.156, *P < .*001). The interpolated ED_50_ for cocaine was 3.04 mg/kg (Table 1).

Administration of METH (black triangles), MDMA (white triangles), mephedrone (white diamonds), and naphyrone (black diamonds) all produced dose-dependent and full substitution for the cocaine training dose (Figure 1, left panel), and the highest dose tested of each drug elicited responding which was significantly different from the discriminative responding elicited by saline (*P < .*001, *t* = 12.752; *t* = 12.752; 11.615; *t* = 10.484; resp.). Morphine (Figure 1, left panel, gray squares) failed to produce responding different from that observed following saline injection, and was tested up to a dose (100 mg/kg) in which response rates were completely suppressed (data not shown). Response rates during all other test sessions were not different from saline. Relative potency to produce cocaine-appropriate responding was METH *>* mephedrone ≈ MDMA ≈ cocaine ≈ naphyrone and ED_50_ values for each drug are presented in Table 1.

## 4. Discussion

In the present studies, near exclusive responding on the cocaine lever occurred following injection of 1.0 mg/kg METH, 3.0 mg/kg mephedrone, and 10.0 mg/kg MDMA, naphyrone, and cocaine, while substitutions with morphine resulted primarily in responding on the saline lever up to a dose (100 mg/kg) where all mice failed to complete the FR10 within the five-minute window. The potency differences among the tested compounds in the present study follow each compound’s transporter selectivity, such that METH (which more selectively inhibits DAT over SERT and also stimulates the release of DA over 5-HT) was the most potent, while the other, relatively nonselective, test compounds had a similar potency to produce cocaine-like responding. This is consistent with the notion that the discriminative stimulus effects of psychostimulants are primarily mediated via interactions with DAT [11,12].

The present data are also consistent with previous reports demonstrating that METH substitutes for cocaine in mice (e.g., [13]), that cocaine substitutes for each enantiomer of MDMA in mice [14], that METH, MDMA, and cocaine substitute for mephedrone in rats [15], and that mephedrone (3.2 mg/kg) and naphyrone (10 mg/kg) substitute for cocaine in rats [9]. Interestingly, 10 mg/kg naphyrone has previously been shown to suppress responding in 33% of rats [9], whereas rates were not systematically decreased by this dose in any mouse in the present study. Also, a recent study in nonhuman primates reported that mephedrone produced cocaine-appropriate responding in only 25% of animals [10]; however mephedrone produced cocaine-like responding in all mice in the present study. As such, the present study suggests species differences in the ability of synthetic cathinones to generalize for cocaine.

In summary, these studies indicate that the synthetic cathinone analogues mephedrone and naphyrone elicit subjective effects in the mouse which are similar to those of the classical psychostimulant cocaine. These abuse-related effects are likely related to the interactions of these compounds with DAT, with naphyrone acting as a passive DA reuptake inhibitor, and mephedrone acting as an amphetamine-like substrate/releaser [4]. These mechanisms likely also underlie the locomotor stimulant effects of these compounds in rodents [9]. As abuse of synthetic cathinones continues, it will be important to continue to investigate the behavioral and pharmacological similarities and differences between these novel psychoactive substances and more well-understood psychostimulants such as cocaine and the amphetamines.

## Figures and Tables

**Table 1:** Structures, pharmacological class, mechanism, relative selectivity for monoamine transporters as inhibitors and substrates, and interpolated ED_50_ values for cocaine-like discriminative stimulus effects. Dashes indicate no pharmacologically-relevant effects for the given endpoint.

Test compound	Structure	Drug class	Mechanism	Inhibition selectivity	Monoamine release	ED_50_ (*±*95% CI)
**(***−***)-cocaine**	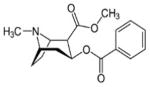	Stimulant	Blocker	DAT ≈ NET ≈ SERT	—	3.0 mg/kg (0.9, 5.2)
**METH**	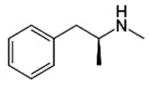	Stimulant	Releaser	NET *>* DAT *>* SERT	DA *>* 5-HT	0.4 mg/kg (0.3, 0.6)
**MDMA**	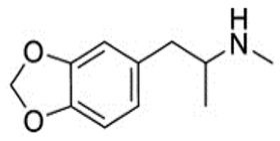	Stimulant	Releaser	NET ≈ SERT ≈ DAT	DA ≈ 5-HT	2.6 mg/kg (0.8, 4.3)
**Mephedrone**	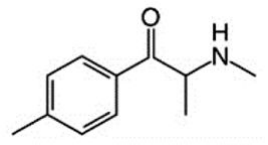	Stimulant	Releaser	NET *>* DAT ≈ SERT	DA ≈ 5-HT	1.5 mg/kg (1.1, 2.0)
**Naphyrone**	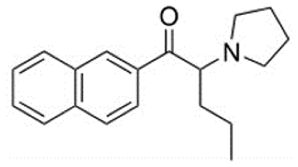	Stimulant	Blocker	NET ≈ DAT ≈ SERT	—	3.2 mg/kg (1.3, 5.0)
**Morphine**	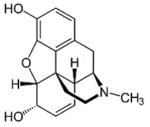	Opioid	*μ* agonist	—	—	—
